# Non-enzymatic Hydrogen Peroxide Sensors Based on Multi-wall Carbon Nanotube/Pt Nanoparticle Nanohybrids

**DOI:** 10.3390/ma7042945

**Published:** 2014-04-10

**Authors:** Zhiying Miao, Di Zhang, Qiang Chen

**Affiliations:** The Key Laboratory of Bioactive Materials, Ministry of Education, College of Life Science, Nankai University, Tianjin 300071, China; E-Mails: mzy@mail.nankai.edu.cn (Z.M.); fluterzd@gmail.com (D.Z.)

**Keywords:** hydrogen peroxide sensor, Pt nanoparticles, multi-wall carbon nanotubes, electrochemical sensor

## Abstract

A novel strategy to fabricate a hydrogen peroxide (H_2_O_2_) sensor was developed by using platinum (Pt) electrodes modified with multi-wall carbon nanotube-platinum nanoparticle nanohybrids (MWCNTs/Pt nanohybrids). The process to synthesize MWCNTs/Pt nanohybrids was simple and effective. Pt nanoparticles (Pt NPs) were generated *in situ* in a potassium chloroplatinate aqueous solution in the presence of multi-wall carbon nanotubes (MWCNTs), and readily attached to the MWCNTs convex surfaces without any additional reducing reagents or irradiation treatment. The MWCNT/Pt nanohybrids were characterized by transmission electron microscope (TEM), and the redox properties of MWCNTs/Pt nanohybrids-modified Pt electrode were studied by electrochemical measurements. The MWCNTs/Pt-modified electrodes exhibited a favorable catalytic ability in the reduction of H_2_O_2_. The modified electrodes can be used to detect H_2_O_2_ in the range of 0.01–2 mM with a lower detection limit of 0.3 μM at a signal-to-noise ratio of 3. The sensitivity of the electrode to H_2_O_2_ was calculated to be 205.80 μA mM^−1^ cm^−2^ at working potential of 0 mV. In addition, the electrodes exhibited an excellent reusability and long-term stability as well as negligible interference from ascorbic acid, uric acid, and acetaminophen.

## 1. Introduction

Hydrogen peroxide is a major messenger molecule in various redox-dependent cellular signaling transductions [1]. It is also known that H_2_O_2_ is abnormally produced in the progress of inflammation by causing oxidative damage [2]. Therefore, sensitive detection of a trace level of H_2_O_2_ is of great importance in health inspection and environment protection [3–6]. Various analytical methods including spectroscopy and electrochemistry have been used to detect H_2_O_2_ [7–9]. In particular, electrochemical techniques based on enzyme-modified electrodes have attracted much interest [10,11]. However, enzyme-modified electrodes usually suffer from high cost, limited lifetime, inherent instability, and complicated immobilization procedure [12]. Consequently, it is imperative to develop non-enzymatic H_2_O_2_ sensors with high sensitivity.

Recent studies indicated that Pt nanoparticles (Pt NPs) exhibited catalytic activity for the reduction of H_2_O_2_ [13–15]. Meanwhile carbon nanotubes (CNTs) have attracted tremendous attention as a result of their ability to promote electron transfer reactions and high thermal stability [16–18]. Therefore, both CNTs [19,20] and Pt NPs [13–15] have been widely employed for detecting H_2_O_2_. In order to take full advantage of these nanomaterials, it is desirable to create novel CNT/Pt nanohybrids, so that the unique properties of each material can be integrated because the interactions between the two components may bring out novel properties.

To fabricate Pt NPs coated MWCNTs, electrochemical, chemical and physical methods have been employed so far [21,22]. However, most of the methods in the synthesis of MWCNTs/Pt NPs nanohybrids are time-consuming and complicated, requiring different kinds of chemicals and templates [23–25].

In this article, by combining the advantages of carbon nanotubes and Pt NPs, MWCNT/Pt NPs nanohybrids were designed and synthesized for the detection of H_2_O_2_. In our protocol, no additional reagent or irradiation was required because Pt NPs were reduced *in situ* onto the MWCNTs, in clear contrast to other reported procedures. The resulted nanomaterials were characterized by transmission electron microscopy (TEM), and the response of the modified electrode to H_2_O_2_ was studied by amperometric measurements. The Pt NPs-modified electrodes showed a high activity in reduction of H_2_O_2_ with a negligible interference from other electroactive molecules. The study can provide a promising platform for fabricating nonenzymatic electrodes and affinity matrix.

## 2. Results and Discussion

### 2.1. Microscopic Observations of MWCNTs-Pt NPs Nanohybrids

**Figure 1.**
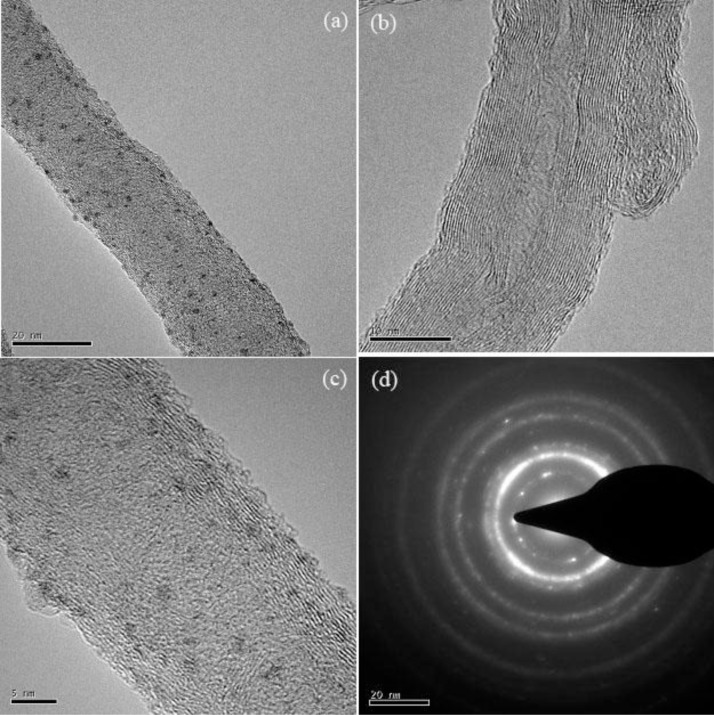
(**a**) TEM images of Pt NPs deposited on MWCNTs; (**b**) MWCNTs without Pt NPs; (**c**) a magnified image for Pt NPs deposited onto the MWCNTs surfaces; (**d**) the selected area electron diffraction (SAED) image of the nanoparticles.

The morphology of nanohybrids was shown in Figure 1. Figure 1(a) shows TEM images of the MWCNTs modified with Pt NPs. Figure 1(c) shows that Pt NPs with a uniform size of about 1–2 nm were deposited onto the MWCNTs surface. In contrast, no such morphology of nanoparticles was observed on the surfaces of unmodified MWCNTs (Figure 1(b)). Figure 1(d) shows the selected area electron diffraction (SAED) image of the nanoparticles, indicating the phase structures of Pt single nanoparticles [26]. Although the spherical structure of Pt NPs decomposed to some degree, the main structure could still be observed on MWCNTs after stored at 4 °C for a month, showing a firm binding of PtNPs on the MWCNTs.

**Figure 2.**
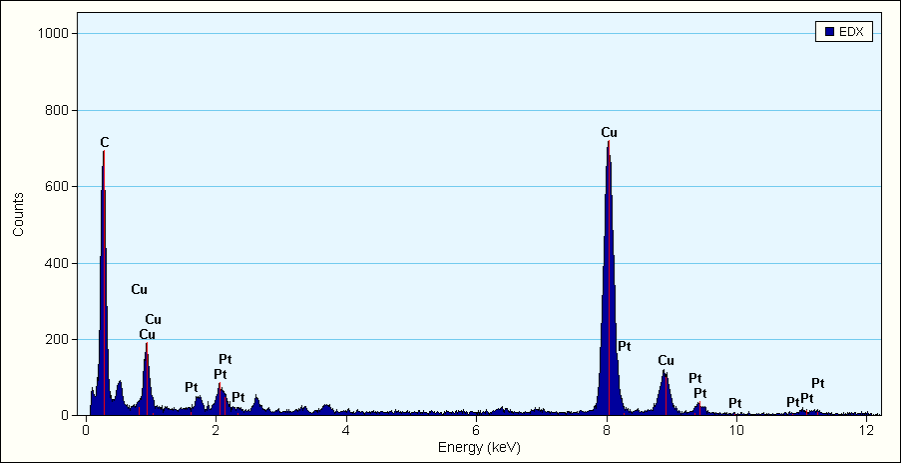
EDX spectrum of the MWCNTs-Pt NPs nanohybrids on copper grids.

Figure 2 shows EDX spectrum, showing that all the samples were of high purity. The EDX shows Pt signals from the nanoparticles; other peaks (C and Cu) were from the copper grids used during the analysis. These results confirm that the Pt NPs have been coated on the MWCNTs.

The numerous carboxylic acid groups on the MWCNTs could play the roles of catalyst, Pt “catcher”, and supporter. In this case, Pt NPs were *in situ* generated from the K_2_PtCl_6_ aqueous solution at room temperature and attached to the convex surfaces of MWCNTs, forming the MWCNTs/Pt NPs nanohybrids. We have tried to use also MWCNTs without carboxylic acid groups to prepare MWCNTs/Pt nanohybrids, but no Pt NPs could be obtained on the surface of the MWCNTs, indicating an essential role of carboxylic acid residues in the reduction of Pt on the surface of MWCNTs.

### 2.2. Electrochemical Response of MWCNTs/Pt Nanohybrids to H_2_O_2_

**Figure 3.**
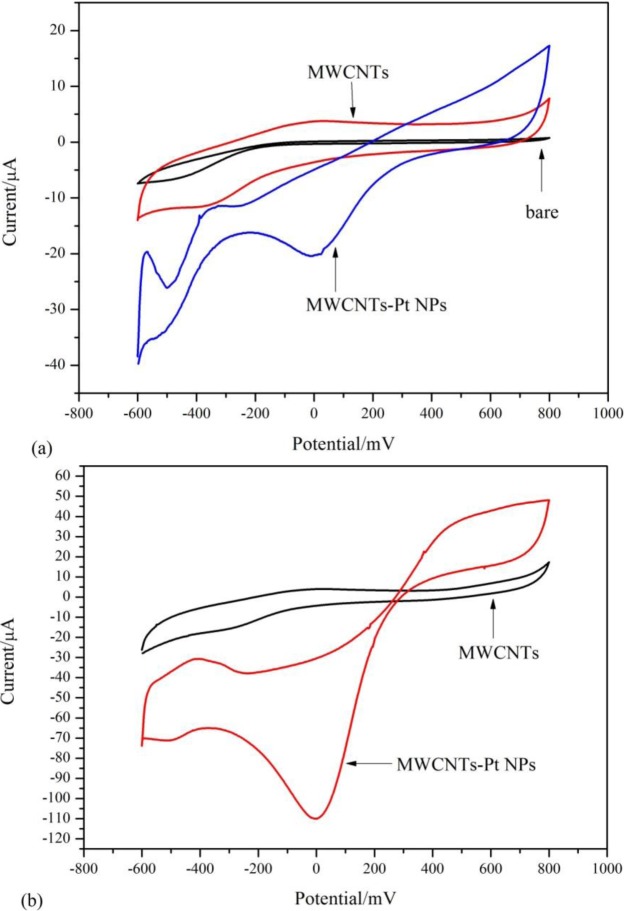
Cyclic voltammograms of bare Pt electrode, MWCNTs/Pt electrode. (**a**) MWCNTs/Pt NPs/Pt electrode in 0.1 M PBS without H_2_O_2_; (**b**) MWCNTs/Pt NPs/Pt electrode in 0.1 M PBS with H_2_O_2_.

Cyclic voltammetry (CV) is often used to estimate the true electroactive surface area of the modified electrode [27,28]. CVs of bare Pt electrode, MWCNTs/Pt electrode, and MWCNTs/Pt NPs/Pt electrode recorded in 0.1 M PBS were illustrated in Figure 3(a). There was no apparent redox process on the bare electrode. Diffusion current clearly increased for the MWCNTs-modified electrode owing to increased catalytically-active surface area. The diffusion current of the MWCNTs/Pt NPs nanohybrids-modified electrode further increased. An electroactive surface area of electrode can be estimated for a reversible and diffusion controlled process according to the Randles-Sevcik equation (Equation (1)) [29], where *Ip* relates to the redox peak current, *A* is the area of the electrode (cm^2^), *n* represents the number of electron participating in the reaction which is equal to 1, *D* is the diffusion coefficient of the molecule in solution which is (6.70 ± 0.02) × 10^−6^ cm^2^ s^−1^, *C* is the concentration of the probe molecule in the solution which is 10 mM and *v* is the scan rate (V s^−1^).

$$]Ip=2.69\times 10^{5}A\;D^{1/2}n^{3/2}v^{1/2}\;C]$$ (1)

According to the above equation, we can obtain the surface area of the electrode. The calculated value of the electroactive surface area for the MWCNTs/Pt NPs nanohybrids-modified electrode was about 3.04- and 2.06-times higher than those of the bare Pt electrode and MWCNTs modified electrodes, respectively. In addition, the combination of the advantages of MWCNTs (large edge plane/basal plane ratio, enhanced conductivity, and rapid electrode kinetics) with well dispersive Pt NPs (high catalytic activity and large surface area) possesses higher electro-active surface areas, which facilitates the adsorption of detection molecules. CVs of the electrodes in the presence of H_2_O_2_ are shown in Figure 3(b). For the MWCNTs-modified electrode, nearly no redox activity is observed for H_2_O_2_. In contrast, CV for the MWCNTs/Pt NPs-modified electrodes changed dramatically, in which the reduction (cathodic) current centered around 0 mV increased. This observation is a clear evidence for the electrocatalysis by Pt NPs. The highest current signal with lower reduction overvoltage are observed for the MWCNTs/Pt NPs modified electrode, which means that the MWCNTs/Pt NPs-modified electrode exhibits the best electrocatalytic activity towards H_2_O_2_ among them. It is likely that the MWCNTs used here as a supporting matrix could well disperse Pt NPs, preventing them from aggregation and thus making them exhibit large active sites to easily contact H_2_O_2_ for the electrocatalytic process.

We can detect H_2_O_2_ at 0 mV as working potential using the MWCNTs/Pt NPs-modified electrode, judging from the CV reported in Figure 3(b). The applied potential for the MWCNTs/Pt NPs-modified electrode is much lower than those for previously-reported H_2_O_2_ sensors [30–32]. Therefore, the background current may be decreased and the response to interference materials can be minimized.

**Figure 4.**
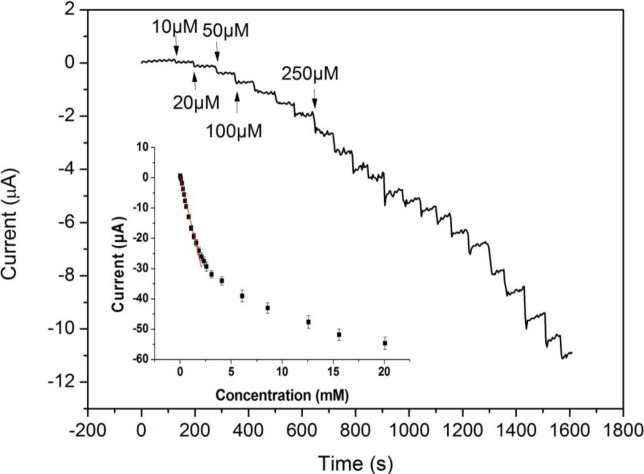
Amperometric responses of the MWCNTs/Pt NPs-modified Pt electrode upon successive addition of H_2_O_2_ in 0.1 M PBS (pH 7.0). Applied potential: 0 mV. Inset: A calibration curve.

The amperometric response of the modified Pt electrode upon successive addition of H_2_O_2_ in PBS (pH 7.0) was studied (Figure 4). When the H_2_O_2_ was added to the PBS solution, the reduction current increased rapidly to reach a steady-state value within 5 s (achieving 95% of the steady-state current). The inset of Figure 4 shows a calibration curve of the H_2_O_2_ sensor. The response was proportional to the H_2_O_2_ concentration in the range from 0.01 to 2.0 mM with a correlation coefficient of 0.997. The sensitivity was 205.80 μA mM^−1^ cm^−2^, which was much higher than the previously reported values [33,34]. In addition, the lower detection limit was 0.3 μM at the signal-to-noise ratio of 3, which was lower than the reported values [32,35]. We have summarized performance characteristics of H_2_O_2_ sensors in Table 1. It is clear that the proposed MWCNTs/Pt NPs/Pt electrode shows better performance than other non-enzymatic H_2_O_2_ sensors. This confirms the excellent performance of MWCNTs/Pt NPs/Pt composite as a material in H_2_O_2_ detection.

The analysis of real samples has also been carried out. Table 2 collects the results for the determination of H_2_O_2_ in disinfected fetal bovine serum (FBS). The recoveries are between 99.8% and 103.4%, indicating that the proposed method can be applied in real sample analysis.

### 2.3. Interference Study

**Figure 5.**
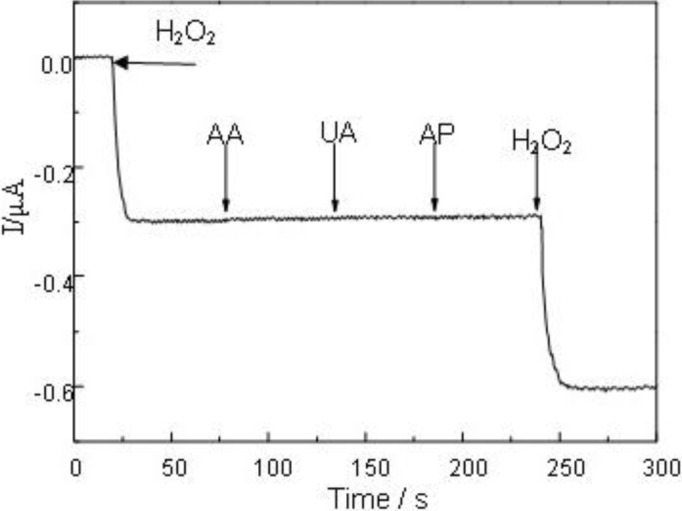
Amperometric responses of the MWCNTs/Pt NPs/Pt electrode upon subsequent additions of 1 mM H_2_O_2_ and 0.1 mM AA, 0.1 mM UA, 0.1 mM AP at 0 mV *vs*. Ag/AgCl.

In real samples, some co-existing electroactive species such as ascorbic acid (AA), uric acid (UA), acetaminophen (AP) might affect the sensor response. The effects of the interference compounds to the H_2_O_2_ sensor were studied by comparing the amperometric responses of the electroactive species (0.1 mM) and H_2_O_2_ (1 mM) at the potential of 0 mV. Figure 5 shows that the successive addition of each interfering species brought out hardly discernible current response. The responses caused by AA, UA and AP could be negligible, and a well-defined H_2_O_2_ response was obtained. The anti-interference ability is largely attributed to the low working potential of 0 mV employed in the determination of H_2_O_2_.

## 3. Experimental Section

### 3.1. Reagents and Materials

Multi-wall carbon nanotubes (30–50 nm diameter and 0.5–1 μm length, with >95% purity, and carboxylic acid groups 0.73 wt%) were obtained from Institute of Organic Chemistry, Chinese Academy of Sciences (ChengDu, China). Hydrogen peroxide (30%, v/v aqueous solution) was obtained from Tianjin Eastern Chemical Reagent Co. Potassium chloroplatinate (K_2_PtCl_6_) was obtained from Tianjin KRS Fine Chemcal Co. Ltd. (Tianjin, China). Uric acid, ascorbic acid and acetaminophen were obtained from Tianjin Damao Chemical Reagent Co. (Tianjin, China). All other reagents were of analytical grade and used without further purification. All aqueous solutions were prepared with Milli-Q deionized water. Phosphate-buffered saline (PBS) was prepared by mixing 25 mL 0.2 M KH_2_PO_4_ aqueous solution with 29.54 mL 0.1 M NaOH and diluted to 100 mL with deionized water. All experiments were performed in PBS at room temperature, approximately 25 °C.

### 3.2. Apparatus and Electrochemical Measurements

The MWCNTs/Pt NPs nanohybrids were characterized with transmission electron microscopy (TEM, Philips T20 microscopy). Electrochemical measurements were carried out in a conventional three-electrode system. The MWCNTs/Pt NPs nanohybrids-modified Pt electrodes (3 mm diameter) were used as a working electrode, with a platinum spiral wire (1 mm diameter) as a counter electrode and an Ag/AgCl electrode (saturated with KCl) as a reference electrode. Amperometric measurements were performed by using Potentiostat-Galvanostat (EG&G PARC Model 283 with a software M270) (USA).

### 3.3. Preparation of H_2_O_2_ Sensor

#### 3.3.1. Modification of Multi-wall Carbon Nanotubes

Pristine MWCNTs were purified under strong sonication in a mixture of concentrated sulfuric and nitric acid 3/1 (v/v) for 4 h. The resultant black suspension was then diluted with water, through the high-speed centrifugal way to remove the supernatant, then dissolved in water to prepare a solution of 1 mg mL^−1^.

#### 3.3.2. Synthesis of MWCNTs/Pt NPs Nanohybrids

1 mL MWCNTs (1 mg mL^−1^) were put into a flask on a magnetic stirrer. While stirring at room temperature, 2 mL of K_2_PtCl_6_ solution (0.01 M) was added dropwise into the flask. The reaction mixture was ultra-sonicated for 2 h and then stirred for 20 h at room temperature. The reaction product (MWCNTs/Pt NPs nanohybrids) was collected by centrifuging.

#### 3.3.3. Electrode Modification with MWCNTs/Pt NPs Nanohybrids

Pt electrode was polished with a chamois leather containing 0.05 μm alumina powders, rinsed thoroughly with doubly distilled water, then immersed into HNO_3_:HCl:H_2_O (V:V:V = 1:3:4) solution for 3 min. The electrode was ultrasonically cleaned in ethanol and doubly distilled water, and dried at room temperature. MWCNTs/Pt NPs nanohybrids solutions (5 μL, 1 mg mL^−1^) were dropped on the surface of the Pt electrode. After drying, the modified electrode was washed and used as an amperometric sensor in phosphate buffer (pH 7.0).

## 4. Conclusions

The main feature of this work was to propose a new non-enzymatic H_2_O_2_ sensor based on MWCNTs/Pt nanohybrids, which was prepared in a fast and simple procedure. In particular, experimental results demonstrated that Pt NPs were able to catalyze the electro-reduction of H_2_O_2_. The modified electrode exhibited an excellent selectivity to H_2_O_2_ in the presence of possible interference compounds, which can be attributed to the relatively lower working potential at 0 mV. The present study may provide a feasible approach to develop new kinds of non-enzymatic amperometric sensors.

## Figures and Tables

**Table 1.** Comparison of performance of H_2_O_2_ sensors.

Electrode	Applied potential (V)	Lower detection limit (μM)	Linear range (mM)	Sensitivity (μA mM^−1^ cm^−2^)	Reference
PtNP/NAE	0.65	1	0.02–20	194.60	[29]
GNS-nPt	0.4	5 × 10^−4^	5 × 10^−7^–12	115.28	[31]
Se/Pt	0	3.1	0.01–15	39.89	[11]
PVA-MWCNTs-PtNPs	0	0.7	0.002–3.8	122.63	[34]
PDDA/t-GO-Pt/GCE	−0.1	0.65	0.001–5	−	[21]
GN-Pt/GCE	0	0.5	0.002–0.71	−	[36]
GO/AuNPs/CS	−0.2	−	0.2–4.2	99.5	[37]
MWCNTs-Pt NPs/Pt	0	0.3	0. 01–2	205.80	This work

**Table 2.** Determination of H_2_O_2_ in disinfected fetal bovine serum (FBS) samples.

Sample^a^	Added (mmol L^−1^)	Found^b^ (mmol L^−1^)	RSD (%, n = 6)	Recovery (%)
1	0.5	0.51	3.2	101.3
2	1.0	0.99	5.1	99.8
3	2.0	2.07	4.5	103.4

a The samples were diluted 100 times;

b Average of six measurements.
